# ﻿Five new species and three new country records of Megaspilidae from Xizang Autonomous Region, China (Hymenoptera, Ceraphronoidea)

**DOI:** 10.3897/zookeys.1260.156058

**Published:** 2025-11-20

**Authors:** Xu Wang, Fang Li, Wen-Jing Zhao, Yi-Xin Huang, Chun-Hui Xiang, Hua-Yan Chen, Chao-Dong Zhu

**Affiliations:** 1 Anhui Provincial Key Laboratory of the Conservation and Exploitation of Biological Resources, Key Laboratory of Biotic Environment and Ecological Safety in Anhui Province, College of Life Sciences, Anhui Normal University, Wuhu, Anhui 241000, China; 2 State Key Laboratory of Animal Biodiversity Conservation and Pest Control: SKLA2501, Institute of Zoology, Chinese Academy of Sciences, Beijing, China; 3 Collaborative Innovation Center of Recovery and Reconstruction of Degraded Ecosystem in Wanjiang Basin Co-founded by Anhui Province and Ministry of Education; School of Ecology and Environment, Anhui Normal University, Wuhu, Anhui 241000, China; 4 State Key Laboratory of Plant Diversity and Specialty Crops and Guangdong Provincial Key Laboratory of Applied Botany, South China Botanical Garden, Chinese Academy of Sciences, Guangzhou 510650, China; 5 College of Biological Sciences/International College, University of Chinese Academy of Sciences, Beijing, China

**Keywords:** Ceraphronoidea, Megaspilidae, new records, new species, taxonomy

## Abstract

Five new species of the Megaspilidae are described from Xizang (or Tibet, SW China): *Conostigmus bomensis* Wang & Zhu, **sp. nov.**, *Conostigmus jilongensis* Wang & Zhu, **sp. nov.**, *Conostigmus shigatsensis* Wang & Zhu, **sp. nov.**, *Conostigmus kairus* Wang & Zhu, **sp. nov.**, and *Dendrocerus tumidulus* Wang & Zhu, **sp. nov.***Lagynodes acuticornis* (Kieffer, 1906), *Conostigmus rufinotum* Dodd, 1914, and *Dendrocerus sergii* Alekseev, 1994 are reported for the first time from China based on specimens collected from Xizang. A key to all known species of Chinese Megaspilidae is included.

## ﻿Introduction

The wasp family Megaspilidae (Hymenoptera, Ceraphronoidea) is comprised of two subfamilies worldwide, Megaspilinae and Lagynodinae. The subfamily Lagynodinae contains 27 described extant and fossil species in six genera: *Aetholagynodes* Dessart, 1994, *Archisynarsis* Szabó, 1973, *Holophleps* Kozlov, 1966, *Lagynodes* Förster, 1841, *Prolagynodes* Alekseev & Rasnitsyn, 1981, and *Typhlolagynodes* Dessart, 1981 (Dessart 1987; Johnson and Musetti 2004). In Lagynodinae, the genus *Lagynodes* was established by Förster (1840), which has the highest species number. Notably, sexual dimorphism is evident in this genus: male forewings are well-developed, with a long radial vein or occasionally reduced, while females are wingless, characterized by an enlarged pronotum and a reduced thorax (Alekseev and Radchenko 2001). There is little information about the host information or ecology of *Lagynodes*. Hosts are documented for only a few species, although these records often lack of detailed biological validation. For instance, *L. pallidus* Boheman, 1832 has been reported as a secondary parasitoid of Braconidae, which parasitizes lepidopteran larvae. However, this association is based on limited observations while species has not yet been thoroughly revised or supported by specific biological studies (Krzyżynski and Ulrich 2015; Moser et al. 2024). Interestingly, some members of subfamily Lagynodinae were often collected in ant nests, suggesting that they parasitize them or live as inquilines in these nests (Salden 2023).

Six species of *Lagynodes* have been recorded from both the Oriental and Palaearctic regions (Johnson and Musetti 2004): *L. acuticornis* (Kieffer), *L. biroi* Szelenyi, *L. occipitalis* Kieffer, *L. ooii* Dessart, *L. pallidus* (Boheman), and *L. thoracicus* Kieffer. Among them, *L. pallidus* has been reported in Taiwan (Dessart 1981). In this paper, we report *L. acuticornis* (Kieffer, 1906) as a new record for the genus *Lagynodes* collected in Xizang.

The subfamily Megaspilinae contains more than 300 valid species belonging to six genera: *Conostigmus* Dahlbom, 1858, *Creator* Alekseev, 1980, *Dendrocerus* Ratzeburg, 1852, *Megaspilus* Westwood, 1829, *Platyceraphron* Kieffer, 1906, and *Trichosteresis* Förster, 1856. Among them, the genera *Conostigmus* and *Dendrocerus* exhibit high numbers of species; there are more than 170 species found in the genus *Conostigmus* worldwide (Bijoy et al. 2014; Mikó et al. 2016, 2018; Trietsch et al. 2020; Cui et al. 2023), with 106 species reported from the Palearctic and the Oriental regions (Dessart 1997; Johnson and Musetti 2004; Fu et al. 2021; Cui et al. 2023). Previous to this manuscript, there were only eight species recorded in China: *C. abdominalis* Boheman, *C. ampullaceus* Dessart, *C. villosus* Dessart, *C. xui* Cui & Wang, *C. quadripetalus* Wang & Chen, *C. electrinus* Wang & Chen, *C. acutus* Wang & Chen, and *C. nankunensis* Qian & Wang (Fu et al. 2021; Cui et al. 2023; Qian et al. 2024; Wang et al. 2024). Species of *Conostigmus* may attack insects in the orders Hymenoptera, Coleoptera, Diptera, Trichoptera, and Mecoptera (Moser et al. 2024).

There are more than 120 species of *Dendrocerus* found worldwide (Mikó et al. 2011), while 48 species reported from the Palearctic and the Oriental regions. Eight species are known from China: *D. angustus* Dessart, *D. carpenteri* Curtis, *D. laticeps* Hedicke, *D. laevis* Ratzeburg, *D. anisodontus* Wang, Chen & Mikó, *D. bellus* Wang, Chen & Mikó, *D. lui* Li & Wang, and *D. amamensis* Takada (Ratzeburg 1852; Rondani 1877; Hedicke 1929; Chen and Huang 1989; Dessart 1999; Wang et al. 2021; Li et al. 2023). Species of *Dendrocerus* comprise of both primary parasitoids and hyperparasitoids at the secondary, tertiary, or even quaternary levels, with hosts such as Hemiptera, Neuroptera, Coleoptera, Diptera, and Hymenoptera (Mikó and Deans 2009; Moser et al. 2024).

The Xizang Autonomous Region, spanning from 26°52’ to 36°32’ north latitude and 78°24’ to 99°06’ east longitude, is situated in southwestern China. It covers an area of ca 1,228,400 square kilometers, accounting for ~12.5% of China’s total territory, and is renowned as the “Roof of the World” (Di et al. 2013). The new species collected in this study were all distributed in Bomi County of Linzhi City in eastern Xizang and Jilong County of Rikaze City in southwestern Xizang. In this paper, we describe five new species and re-describe three newly recorded species of the Megaspilidae from Xizang, China.

## ﻿Materials and methods

Specimens were obtained from sweep nets, Malaise traps, and yellow pan traps. Each specimen was placed in a 1.5-mL centrifuge tube filled with 1 mL of ethanol and stored at -20 °C. Specimens are deposited in the Insect Collection of Anhui Normal University (**AHNU**), Wuhu, China.

Specimens were positioned and stabilized for imaging using molding clay. Images of dried specimens were taken with a Leica M205A stereomicroscope (Leica Microsystems CMS GmbH, Wetzlar, Germany), equipped with a Leica DFC-500 digital camera. Image stacking was performed with Leica Microsystems CMS GmbH and extended focus images were annotated and modified with Adobe Photoshop Version 2020 using Image/Adjustments/Levels mask and Image/Adjustments/Sharpness tools. Map creation involved drawing and retouching using Adobe Photoshop v. 2020 software.

The metasoma was removed from the specimens and placed in 35% H_2_O_2_ for 24 h, followed by 5% acetic acid for 24 h, and rinsing in distilled water for 1 h, then transferred to a droplet of glycerin on a concavity slide. Dissections were performed in glycerin using #5 forceps and #2 insect pins. The dissected genitalia were stored in glycerin for further analysis. Measurements are given in microns. Genitalia terminology follows the Hymenoptera Anatomy Ontology. Abbreviations and morphological terms follow Mikó and Deans (2009) and Trietsch et al. (2020).

Abbreviations, morphological terms (Table 1), and genitalic terminology follow Mikó and Deans (2009). Measurements are given in microns.

**Table 1.** Abbreviations and morphological terms used in text.

Ascw	Anterior mesoscutal width
**A1, A2, ..., A5**	Branches on the Flagellomere 1, 2, ..., 5
**EHf**	Eye height, frontal view
**F1, F2, ..., F9**	Flagellomere 1, 2, ..., 9
**HH**	Head height, anterior view
**HL**	Head length
**HW**	Head width
**IOS**	Interorbital space
**LOL**	Lateral ocellar length, shortest distance between inner margins of median and lateral ocelli
**OOL**	Ocular ocellar length, minimum distance between a posterior ocellus to the eye margin
**POL**	Posterior ocellar length, shortest distance between inner margins of posterior ocelli
**Pscw**	Posterior mesoscutal width

## ﻿Results

**Family MEGASPILIDAE**

### ﻿Key to species of Megaspilidae from China

**Table:** 

1	Forewings of males without pterostigma, only with stigmal vein; second syntergum syntergite with ≤3 distinct longitudinal carinae at its base; mesoscutum of females without longitudinal furrows (Subfamily Lagynodinae)	**2**
–	Forewings of males and females with pterostigma; second syntergum syntergite with numerous distinct longitudinal carinae at its base; mesoscutum of females with longitudinal median furrow and notauli or at least with trace of notauli (Subfamily Megaspilinae)	**3**
2	Female with F9 acutely pointed distally, median process on intertorular carina absent; prothorax rounded, posterior margin concave, metathorax absent; 5 syntergum carinae, reaching 2/5 of syntergum	***L. acuticornis* (Kieffer, 1906)**
–	Female with F9 obtuse (or blunt) distally, median process on intertorular carina acute; prothorax oval-shaped, posterior concave cylindrical, metathorax short anterior margin concave; 3 syntergum carinae, reaching 3/5 of syntergum	***L. pallidus* (Boheman, 1832)**
3	POL shorter than or equal to OOL, sternaulus present or absent, wings present or absent, male genitalia parossiculi never fused with gonostipes, head globular or circular in shape (*Conostigmus* Dahlbom)	**4**
–	POL longer than OOL, sternaulus absent, wings never absent, male genitalia parossiculi fused with gonostipes, head triangular in shape (*Dendrocerus* Ratzeburg)	**16**
4	Mesosoma ≥1.8 × longer than wide, with a long neck	***C. ampullaceus* Dessart, 1997**
–	Mesosoma at most 1.6 × longer than wide, mesosoma without a long neck	**5**
5	Facial pit absent	**6**
–	Facial pit present	**8**
6	Facial sulcus present	**7**
–	Facial sulcus absent	***C. quadripetalus* Wang & Chen, 2024**
7	Harpe spatulate or spoon-shaped and longer than the gonostipes in lateral view	***C. abdominalis* Boheman, 1832**
–	Harpe slightly shorter than gonostipes in lateral view and exhibits an inwardly concave at the distal end in lateral view	***C. nankunensis* Qian & Wang, 2024**
8	Anteromedian projection of the metanoto-propodeo-metapecto-mesopectal complex present	**9**
–	Anteromedian projection of the metanoto-propodeo-metapecto-mesopectal complex absent	**14**
9	Preoccipital furrow short, not reaching the line connecting the 2 posterior ocelli	**10**
–	Preoccipital furrow long, reaching the line connecting the 2 posterior ocelli	**12**
10	Three syntergum at most reaching 1/7 length of syntergum	***C. bomensis* Wang & Zhu, sp. nov.**
–	Three syntergum at least reaching 1/4 length of syntergum	**11**
11	Sternaulus length: mesopleuron length 0.41	***C. rufinotum* Dodd, 1914**
–	Sternaulus length: mesopleuron length 0.75	***C. jilongensis* Wang & Zhu, sp. nov.**
12	Basal syntergum carinae reaching 1/4 of syntergum length	***C. shigatsensis* Wang & Zhu, sp. nov.**
–	Basal syntergum carinae reaching 1/3 of syntergum length	**13**
13	Sternaulus length: mesopleuron length ratio 0.6–0.7	***C. xui* Cui & Wang, 2023**
–	Sternaulus length: mesopleuron length ratio 1	***C. kairus* Wang & Zhu, sp. nov.**
14	Basal syntergum carinae reaching 1/4 of syntergum length	***C. acutus* Wang & Chen, 2024**
–	Basal syntergum carinae reaching 1/3 of syntergum length	**15**
15	Head, mesosoma and F1–F9 black, scape pale-colored, the rest of antennae black; pterostigma length vs width 2	***C. villosus* Dessart, 1997**
–	Head and mesosoma reddish brown, metasoma and antennae amber; pterostigma length vs width 3.7–4.5	***C. electrinus* Wang & Chen, 2024**
16	Flagellomeres branched or cylindrical	**17**
–	Flagellomeres toothed	**20**
17	Antennae with 5 branches	**18**
–	Antennae with 6 branches	**19**
18	The fifth branch of the antenna shorter than F6	***D. sergii* Alekseev, 1994**
–	The fifth branch of the antenna longer than F6	***D. angustus* Loomis, 1941**
19	The sixth branch of the antenna shorter than F7	***D. amamensis* Takada, 1974**
–	The sixth branch of the antenna equal to F7	***D. anisodontus* Wang, Chen & Mikó, 2021**
20	Mesoscutum with notauli incomplete	**21**
–	Mesoscutum with notauli complete	**22**
21	Radius shorter than pterostigma	***D. bellus* Wang, Chen & Mikó, 2021**
–	Radius longer than pterostigma	***D. laevis* (Ratzeburg, 1852)**
22	Facial pit absent	***D. carpenteri* (Curtis, 1829)**
–	Facial pit present	**23**
23	Notauli without angle or slightly angled and not connected posteriorly	***D. lui* Li & Wang, 2024**
–	Notauli strongly angled, convergent, and connected posteriorly	**24**
24	Syntergum carinae absent	***D. tumidulus* Wang & Zhu, sp. nov.**
–	Syntergum carinae present	***D. laticeps* (Hedicke, 1929)**

### ﻿Taxonomic account

#### ﻿ Conostigmus

Taxon classificationAnimaliaHymenopteraMegaspilidae

Dahlbom, 1858

14459038-CDFE-5C00-9C75-9D3552930462

Conostigmus Dahlbom, 1858: 291.
Dichogmus Thomson, 1858: 287.
Eumegaspilus Ashmead, 1888: 48.
Eumegalospilus Schulz, 1906: 152.
Conostigmoides Dodd, 1914: 88.
Ecnomothorax Dessart & Masner, 1965: 276.
Dolichoceraphron Hellén, 1966: 15.
Szelenyides Dessart, 1974: 43.

##### Diagnosis.

Antennae with 11 segments in both sexes. Male flagellomeres symmetrical and cylindrical. POL shorter than OOL. Wings present or absent; pterostigma present. Notauli only slightly and steeply angulate anteriorly; anteromedian projection of the metanoto-propodeo-metapecto-mesopectal complex present (not bifurcated) or absent. Male parossiculi independent or fused, and each parossiculus with gonostipes not fused. Sternaulus present or absent.

#### ﻿ Conostigmus rufinotum

Taxon classificationAnimaliaHymenopteraMegaspilidae

Dodd, 1914

A88952AC-1CB4-5840-B446-7860C494E92A

Figs 1, 2

Conostigmus rafthotum Dodd, 1914: 91.
Conostigmus leai Dodd, 1914: 92.
Conostigmus muscosus Dodd, 1914: 92.
Conostigmus tasmunicus Dodd, 1914: 92.
Conostigmus giraulti Dodd, 1914: 93.
Conostigmus pretiosus Dodd, 1915: 454.

##### Material examined.

• 2♂♂ (AHNU), **China**: Xizang, Jilong, Rikaze, D. Wu leg., XZ-25, XZYG-8; • 1♂ (AHNU), **China**: Jilong, Rikaze, 3 Aug. 2021, D. Wu leg., XZ-34.

##### Diagnosis.

This species can be separated from other *Conostigmus* species by the following characters: harpe gradually tapering (distoventral margin of harpe flat, distodorsal margin of harpe concave, in lateral view) , harpe shorter than gonostipes in lateral view, gonossiculus with two spines apically. Proximal end of dorsomedian conjunctiva of the gonostyle-volsella complex blunt. Gonostyle-volsella complex with medioventral ridge absent, gonostyle-volsella complex with length equal that of the gonostipes. Proximal margin of S9 acute; preoccipital furrow structured, not reaching anterior ocellar; notauli and median mesoscutal sulcus very distinct, with continuous fovea; anterior mesopleural sulcus distinct, with continuous fovea; pterostigma 2.4× as long as wide; mesometapleural sulcus distinct, with continuous fovea, mesopleuron 2.4× as long as the sternaulus.

##### Description.

**Male.** Body length: 1.7–1.8 mm.

**Figure 1.**
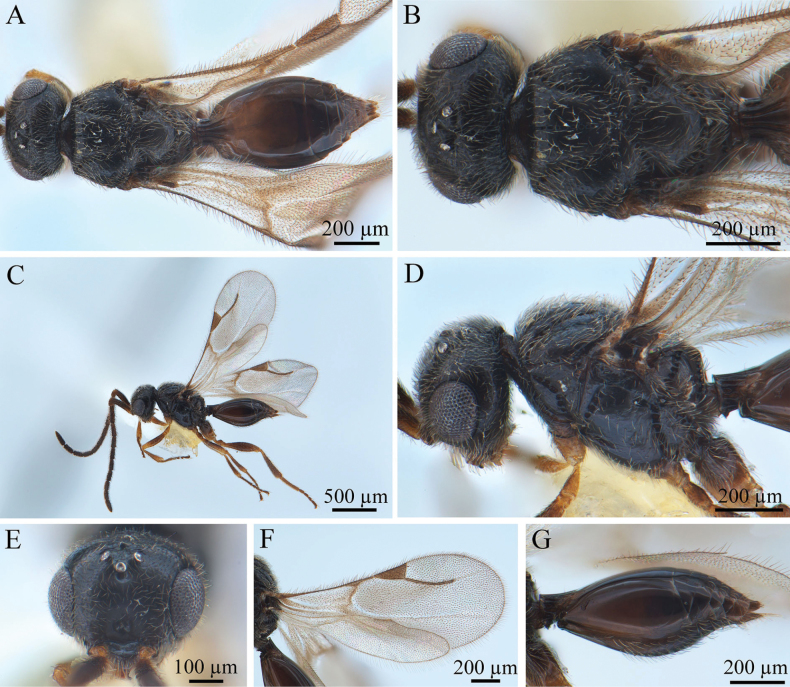
*Conostigmus rufinotum* Dodd, 1914, male. **A.** Dorsal habitus; **B.** Head and mesosoma, dorsal view; **C.** Lateral habitus; **D.** Head and mesosoma, lateral view; **E.** Head, anterior view; **F.** Wings; **G.** Metasoma, lateral view.

***Coloration*** (Fig. 1C). Head, mesosoma, and metasoma black. Scape and flagellum dark brown, pedicel brown, with other antennal segments black. Mouthparts brown; eyes silvery; ocelli silvery-black. Legs usually yellow, sometimes brown proximally, especially on middle sides of femora and tibiae. Syntergum dark brown to black. Pterostigma and vein pale brown. Body pubescence white; marginal fringes of wings brown.

***Antennae*** (Fig. 1C). Scape nearly 4× longer than wide, pedicel small and almost droplet-shaped. Male scape length vs pedicel length 4: 1. Scape length vs F1 length 1.2–1.4. F1 length vs F2 length: 1.2–1.3. F1 longest. F6–F8 (all equal lengths) shortest. Setae short, reaching 1/3 of flagellomere width.

***Head*** (Fig. 1D, E). Head width same as mesosoma. HH: HL = 1.4–1.6. HW: IOS = 1.6–1.7. HW: HH = 1.1–1.2. POL: OOL = 0.6–0.7. Ocellar triangle with short base, OOL: LOL = 2.9–3.0. Head circular in anterior view. Facial pit present, facial sulcus absent. Preocellar pit present, ocellar fovea present. Preoccipital lunula present. Preoccipital furrow present, reaching anterior ocellar. Preoccipital carina present. Upper margins of scrobes V-shaped, intertorular carina absent. Head with dense hairs.

***Mesosoma*** (Fig. 1B, D). Mesosoma narrow, 1.1× longer than wide (length/width/height = 540/480/390 µm); densely pubescent, alutaceous in sculpture; mesoscutum length/width = 230/480 µm, mesoscutum 2.1× wider than long, Ascw/Pscw = 340/370 µm; notauli and median mesoscutal sulcus very distinct, with deep continuous fovea, length equal to width; scutellum width almost equal to length (length/width = 240/250 µm); scutoscutellar sulcus foveolate, single fovea of the scutoscutellar sulcus length equal to width, continuous with interaxillar sulcus. Axilla width longer than length. Pronotum triangular. Anterior mesopleural area present; anterior mesopleural sulcus distinct, with continuous fovea; posteroventral area (part of mesopleuron) smooth, with sparse setae; mesometapleural sulcus distinct, with continuous fovea, in contact with mesopleural pit, mesopleural pit expanded; ventral division of metapleuron smooth with sparse setae; pleural carina with long bristles. The mesopleuron 2.4× as long as the sternaulus. Anteromedian projection of the metanoto-propodeo-metapecto-mesopectal complex present.

***Wings*** (Fig. 1F). Forewing length 1.6 mm, with pterostigma, stigmal vein; hyaline, densely pubescent and marginal fringes numerous. Pterostigma length/width = 220/90 µm, 2.4× as long as wide, triangular, posterior margin (part of pterostigma) straight. Stigmal vein 350 µm, slightly curved in the latter section and 1.6× longer than pterostigma. Hindwing with some transparent veins.

***Metasoma*** (Fig. 1A, G). Metasoma 1.9× longer than wide (length/width/height = 800/410/330 µm). Syntergum smooth, reaching 3/5 of metasoma length. Syntergum with three distinct gastral carinae, reaching 1/3 of syntergum length. Syntergal translucent patch transverse, elliptical in shape.

**Figure 2.**
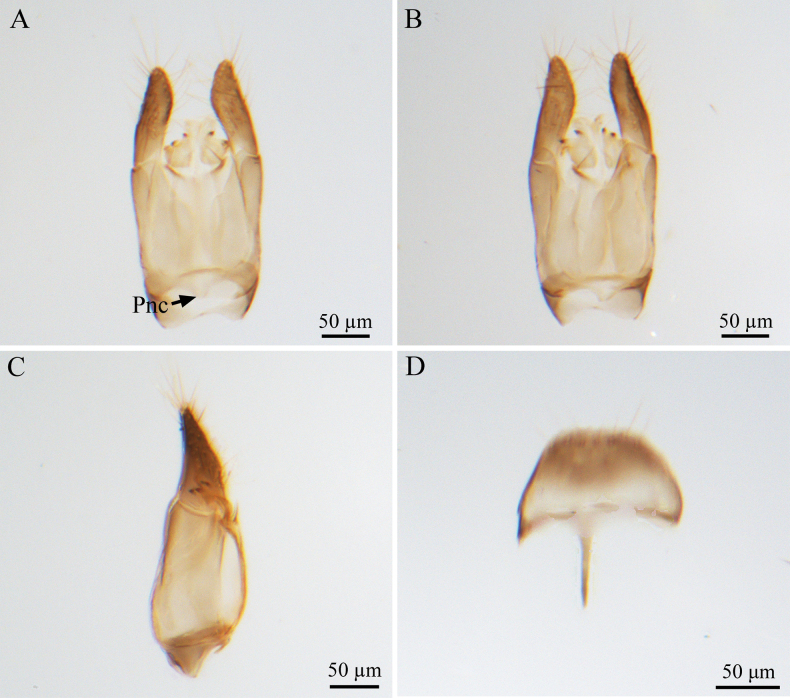
*Conostigmus rufinotum* Dodd, 1914, male, genitalia. **A.** Dorsal view; **B.** Ventral view; **C.** Lateral view; **D.** S9. Abbreviation: Pnc: Proximoventral median projection of cupula.

***Male genitalia*** (Fig. 2). Genitalic cupula present, proximodorsal notch of cupula blunt; distodorsal margin of cupula straight. Cupula <1/2 length of gonostyle-volsella complex in lateral view. Proximoventral median projection of cupula present. Harpe gradually tapering (distoventral margin of harpe flat, distodorsal margin of harpe concave, in lateral view); harpe shorter than gonostipes in lateral view, reaching 3/5 of gonostipes; harpe with more than 17 setae. Proximal end of dorsomedian conjunctiva of the gonostyle-volsella complex blunt. Gonostyle-volsella complex with medioventral ridge absent, gonostyle-volsella complex length half that of the gonostipes. Gonostipes longer than width; parossiculus separated from gonostipes. Parossiculus with one seta apically. Gonossiculus with two spines apically in lateral view. Penisvalva hyaline. S9 umbrella-shaped, with single transverse row of seven setae; submedial projections on proximal margin of S9 absent. Distal margin of male S9 with projections. Proximolateral corner of male S9 without projections, margin smooth. Proximal margin of S9 acute. Medial projections on proximal margin of S9 present, length equal to the length of S9 shield.

**Female.** Unknown.

##### Distribution.

China (Xizang) – new record for China, Australia.

#### ﻿ Conostigmus bomensis

Taxon classificationAnimaliaHymenopteraMegaspilidae

Wang & Zhu sp. nov.

3404D514-70C0-5BD1-89FA-E6749A87F4A8

https://zoobank.org/92F02EB0-07A0-416E-B2EE-F4CD966E3FE6

Figs 3, 4

##### Type material.

***Holotype***: • 1♂ (AHNU), **China**: Xizang, Bomi, Linzhi, 20 Apr. 2021, D. Wu leg., XZ-15. ***Paratype***: • 1♂ (AHNU), **China**: same collection information as preceding, XZYG-4.

##### Diagnosis.

This new species can be separated from other *Conostigmus* species by the following characters: harpe of male genitalia strip-shaped in lateral view (distoventral and distodorsal margins of harpe with projections in lateral view), almost equal to gonostipe length in ventral view, gonossiculus with three spines apically. Proximal margin of S9 without projections. Preoccipital furrow structured, groove, not reaching anterior ocellus; posteroventral area and part of mesopleuron smooth; pterostigma 2.3× as long as wide; mesometapleural sulcus present, groove extending to mesopleural pit; sternaulus 0.45× the length of mesopleuron.

##### Description.

**Male.** Body length: 1.65–1.74 mm.

**Figure 3.**
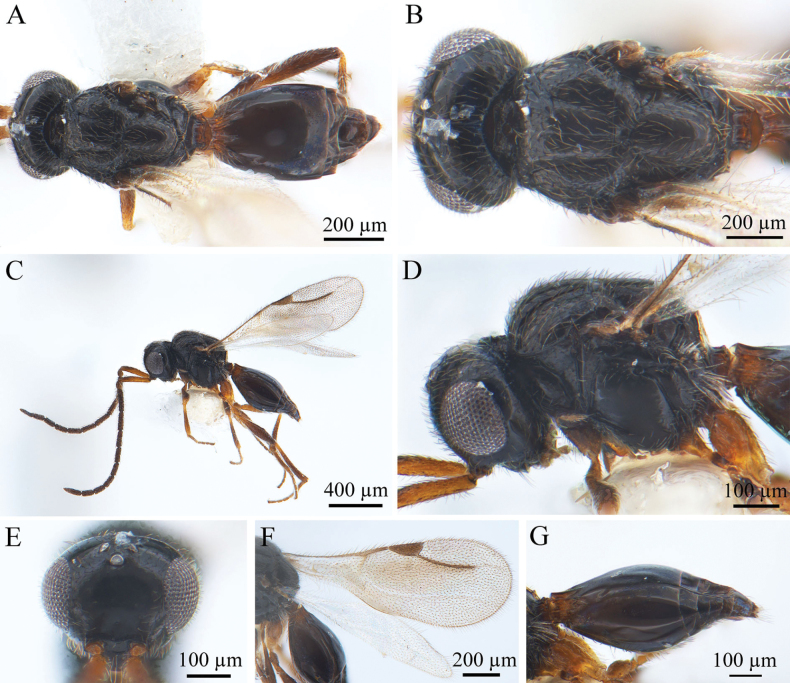
*Conostigmus bomensis* Wang & Zhu, sp. nov., male, holotype. **A.** Dorsal habitus; **B.** Head and mesosoma, dorsal view; **C.** Lateral habitus; **D.** Head and mesosoma, lateral view; **E.** Head, anterior view; **F.** Wings; **G.** Metasoma, lateral view.

***Coloration*** (Fig. 3A, B). Head, mesosoma and metasoma black. Flagellum dark brown, scape and pedicel brown. Mouthparts brown; eyes silvery; ocelli silvery-black. Legs usually brown, sometimes darkened distally especially on middle sides of femora and tibiae. Pterostigma and stigmal vein pale brown. Body pubescence white; margin of wings brown.

***Antennae*** (Fig. 3C). Scape ~4 × longer than wide, pedicel small and rounded. Scape 3.7–3.9× as long as pedicel. Scape length vs F1 length 1.2–1.4. F1 length vs F2 length 1.2–1.4. Longest flagellomere F1. Shortest flagellomere F8. Setae short, length not reaching 1/3 of flagellomere width.

***Head*** (Fig. 3D, E). Head as wide as mesosoma. HH: HL = 1.3–1.5. HW: IOS = 1.5–1.7. HW: HH = 1–1.2. POL: OOL = 0.6–0.8. Ocellar triangle with short base, OOL: LOL = 2.0–2.3. Head circular in anterior view. Facial pit present and shallow, facial sulcus absent. Preocellar pit absent, ocellar fovea present. Preoccipital lunula present. Preoccipital furrow present, grooved, not reaching posterior ocellar. Preoccipital carina present. Upper margins of scrobes V-shaped, intertorular carina present. Head with sparse hairs.

***Mesosoma*** (Fig. 3B, D). Mesosoma somewhat narrow, 1.3× longer than wide (length/width/height = 660/520/430 µm); densely pubescent, alutaceous in sculpture; mesoscutum length/width = 260/520 µm, Ascw/Pscw = 370/400 µm; notauli and median mesoscutal sulcus very distinct, grooved. Scutellum width almost equal to length, length/width = 280/260 µm; scutoscutellar sulcus foveolate; single fovea of the scutoscutellar sulcus longer than width. Axilla width slightly longer than length. Pronotum triangular. Posteroventral area and part of mesopleuron smooth; mesometapleural sulcus present, grooved, extending to mesopleural pit, mesopleural pit expanded; ventral division of metapleuron smooth; pleural carina with long bristles. Sternaulus present, 0.45× the length of mesopleuron; anteromedian projection of the metanoto-propodeo-metapecto-mesopectal complex present.

***Wings*** (Fig. 3F). Forewing length 1.4 mm, with pterostigma, stigmal vein. Hyaline, densely pubescent, and marginal fringes numerous. Pterostigma (length/width = 210/90 µm) 2.3× as long as wide, semi-elliptical, posterior margin convex. Stigmal vein 310 µm, slightly curved in the latter section and 1.5× longer than pterostigma.

***Metasoma*** (Fig. 3A, G). Metasoma 2× longer than wide (length/width/height = 730/490/300 µm). Syntergum smooth, reaching 3/5 of metasomal length. Syntergum with three equal gastral carinae, reaching 3/20 of syntergum length. Syntergal translucent patch present, elliptical.

**Figure 4.**
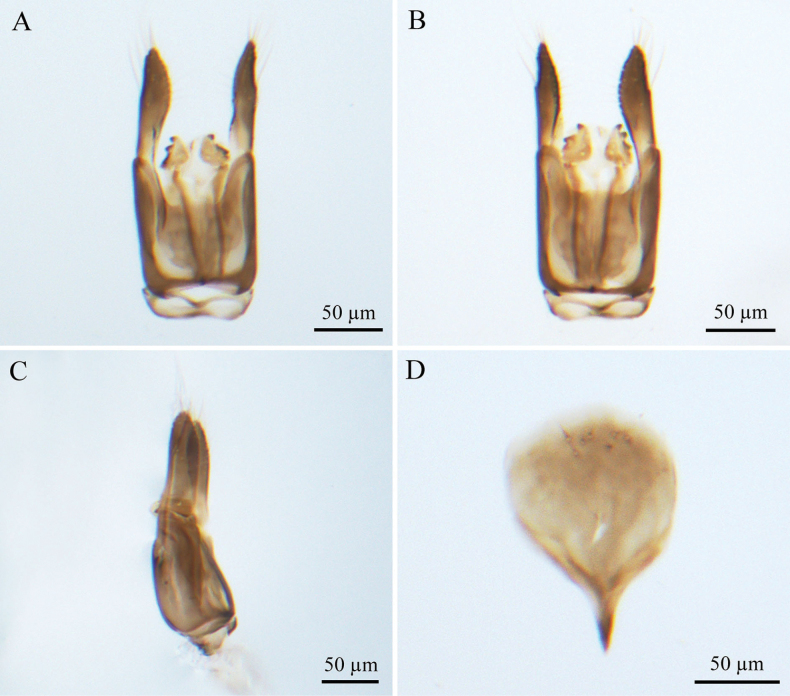
*Conostigmus bomensis* Wang & Zhu, sp. nov., male, holotype, genitalia. **A.** Dorsal view; **B.** Ventral view; **C.** Lateral view; **D.** S9.

***Male genitalia*** (Fig. 4). Genitalic cupula present, proximodorsal notch blunt, distodorsal margin straight. Cupula length <1/2 gonostyle-volsella complex length in lateral view. Proximoventral median projection of cupula present. Harpe strip-shaped in lateral view (distoventral and distodorsal margins of harpe with projections, in lateral view), with pointed tip; length almost equal to gonostipes length in ventral view. Proximal end of dorsomedian conjunctiva of the gonostyle-volsella complex straight. Gonostyle-volsella complex with medioventral ridge absent, gonostyle-volsella complex with length reaching 2/3 that of the gonostipes. Gonostipes longer than width; parossiculus separated from gonostipes. Parossiculus with one seta apically. Gonossiculus with three spines apically in lateral view. Penisvalva straight. S9 blunt, with irregular row of seven pits; submedial projections on proximal margin of S9 absent. Distal margin of male S9 with projections. Proximolateral corner of male S9 with projections, but not acute. Proximal margin of S9 without projections. Medial projections on proximal margin of S9 present, length <1/2 length of S9 shield.

**Female.** Unknown.

##### Etymology.

The species is named after Bomi County, where the species was collected.

##### Distribution.

**China** (Xizang).

##### Remarks.

This species is similar to *C. jilongensis*, but can be distinguished by the strip-shaped harpe with its length approximately equal to the gonostipes in lateral view (vs trapezoidal and ~3/5 the length of gonostipes in *C. jilongensis*), the gonossiculus possessing three apical spines (vs two in *C. jilongensis*), and the absence of a medioventral ridge on the gonostyle-volsella complex (present in *C. jilongensis*).

#### ﻿ Conostigmus jilongensis

Taxon classificationAnimaliaHymenopteraMegaspilidae

Wang & Zhu sp. nov.

EBE658CD-549F-5E0D-B32D-030501F83013

https://zoobank.org/16DD7D9B-A530-4F8E-89E7-3D449761175A

Figs 5, 6

##### Type material.

***Holotype***: • 1♂ (AHNU), **China**: Jilong, Rikaze; 3 Aug. 2021, D. Wu leg., XZ-20. ***Paratypes***: • 4♂♂ (AHNU), **China**: Xizang, Jilong, Rikaze, 3 Aug. 2021, D. Wu leg., XZ-35, XZ-37, XZ-41, XZ-40; • 1♂ (AHNU), **China**: Xizang, Jilong, Rikaze, 3 Aug. 2021, D. Wu leg., XZS-8; • 1♂ (AHNU), **China**: Xizang, Jilong, Rikaze, 17 Apr-23 May. 2021, D. Wu leg., XZL-4.

##### Diagnosis.

This new species can be separated from other *Conostigmus* species by the following characters: harpe of male genitalia trapezoidal in lateral view (proximoventral margin of harpe with projections in lateral view, distodorsal margin of harpe with projections in other species), length shorter than gonostipes length in lateral view; gonossiculus with two spines apically. Gonostyle-volsella complex with medioventral ridge present. Submedial projections on proximal margin of S9 present. S9 shield width: length = 1.6; distal part of S9 shield darker brown, proximal part translucent and paler in color. Dorsal side of gonostipes flat. Preoccipital furrow not reaching anterior ocellus, notauli and median mesoscutal sulcus very distinct, grooved, pterostigma 3× as long as wide; sternaulus 0.75× as long as the mesopleuron.

##### Description.

**Male.** Body length: 1.3–1.5 mm.

**Figure 5.**
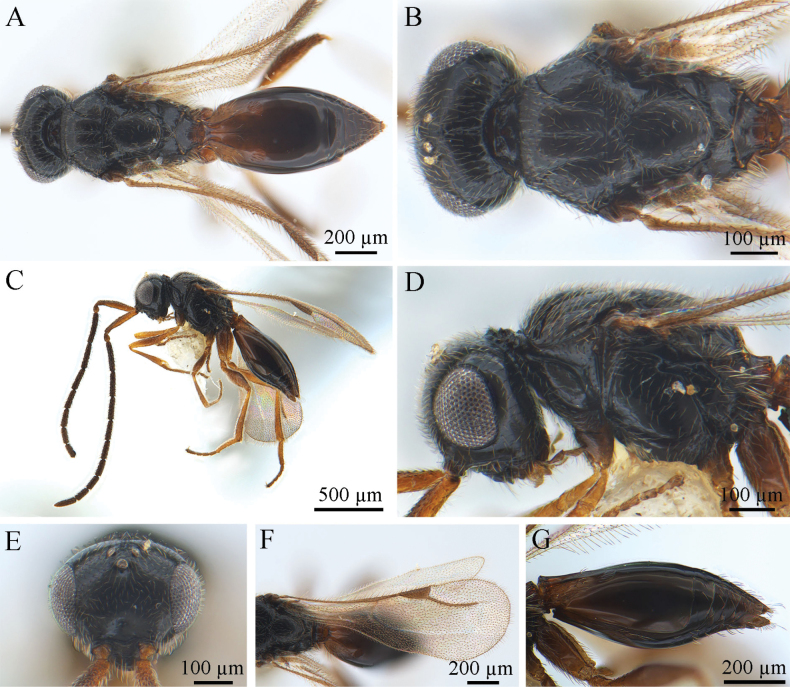
*Conostigmus jilongensis* Wang & Zhu, sp. nov., male, holotype. **A.** Dorsal habitus; **B.** Head and mesosoma, dorsal view; **C.** Lateral habitus; **D.** Head and mesosoma, lateral view; **E.** Head, anterior view; **F.** Wings; **G.** Metasoma, lateral view.

***Coloration*** (Fig. 5C). Head, mesosoma, and metasoma black. Flagellum dark brown, scape and pedicel brown. Mouthparts brown; eyes silvery; ocelli silvery-black. Legs usually brown, sometimes brown proximally, sometimes darkened proximally, especially on middle sides of femora and tibiae. Syntergum dark brown to black. Pterostigma and stigmal vein pale brown. Body pubescence white; marginal fringes of wings brown.

***Antennae*** (Fig. 5C). Scape ~4 × longer than wide, pedicel small and rounded. Male scape length vs pedicel length 4.3–5.4. Scape length vs F1 length 1.1–1.5. F1 length vs F2 length 1.2–1.3. F1 longest. F8 shortest. Setae short, not reaching 1/3 of flagellomere width.

***Head*** (Fig. 5D, E). Head width same as mesosoma. HH: HL = 1.2–1.5. HW: IOS = 1.5–1.6. HW: HH = 1.1. POL: OOL = 0.5. Ocellar triangle with short base, OOL: LOL = 2.0–2.9. Head circular in anterior view. Facial pit present, facial sulcus absent. Preocellar pit present, ocellar fovea present. Preoccipital lunula present. Preoccipital furrow present, not reaching posterior ocellar, fovea (part of preoccipital furrow) length longer than width. Preoccipital carina present. Upper margins of scrobes V-shaped, intertorular carina absent. Head with dense hairs.

***Mesosoma*** (Fig. 5B, D). Mesosoma narrow, 1.5× longer than wide, length/width/height = 560/360/380 µm; densely pubescent, alutaceous in sculpture; mesoscutum length/width = 180/320 µm, mesoscutum 1.8× wider than long, Ascw/Pscw = 230/280 µm; notauli and median very distinct, grooved. Scutellum width almost equal to length, length/width = 210/190 µm; scutoscutellar sulcus foveolate; single fovea of the scutoscutellar sulcus length equal to width, continuous with interaxillar sulcus. Axilla width slightly longer than length. Pronotum triangular. Anterior mesopleural area present; anterior mesopleural sulcus grooved; posteroventral area (part of mesopleuron) smooth, with sparse setae; mesometapleural sulcus present, extending to mesopleural pit, mesopleural pit expanded; ventral division of metapleuron smooth with sparse setae; pleural carina with long bristles. Sternaulus present, 0.75× as long as mesopleuron. Anteromedian projection of the metanoto-propodeo-metapecto-mesopectal complex present.

***Wings*** (Fig. 5F). Forewing length 1.4 mm, with pterostigma and stigmal vein. Hyaline, densely pubescent, and marginal fringes numerous. Pterostigma length/width = 180/60 µm, 3× as long as wide, triangular, posterior margin (part of pterostigma) straight. Stigmal vein 290 µm, slightly curved in the latter section and 1.6× longer than the pterostigma.

***Metasoma*** (Fig. 5A, G). Metasoma 2.2× longer than wide (length/width/height = 790/350/290 µm). Syntergum smooth, reaching 2/3 of metasoma length. Syntergum with three distinct gastral carinae, reaching 1/3 of syntergum length.. Syntergal translucent patch present, elliptical.

**Figure 6.**
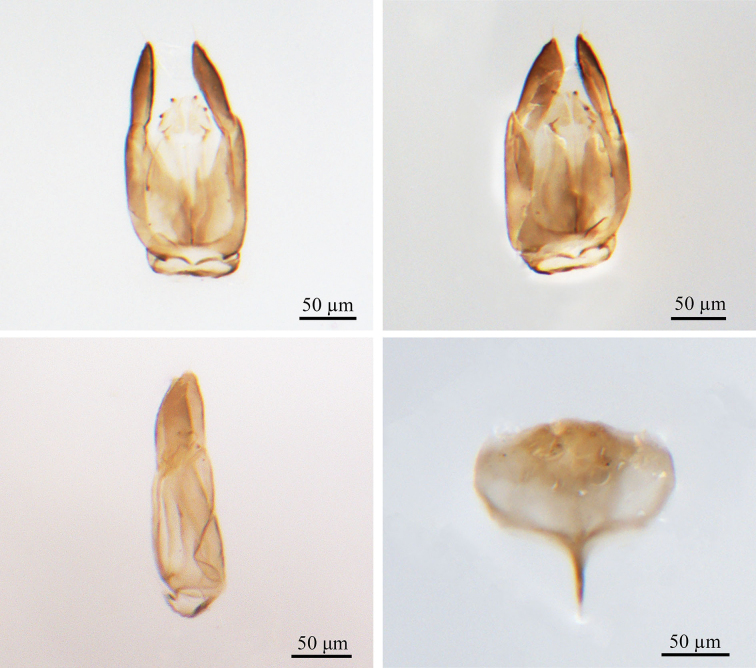
*Conostigmus jilongensis* Wang & Zhu, sp. nov., male, holotype, genitalia. **A.** Dorsal view; **B.** Ventral view; **C.** Lateral view; **D.** S9.

***Male genitalia*** (Fig. 6). Genitalic cupula present, proximodorsal notch of cupula blunt, with a darker brown coloration near the central region; distodorsal margin of cupula straight. Cupula length vs Gonostyle-volsella complex length: cupula <1/2 the length of gonostyle-volsella complex in lateral view. Proximoventral median projection of cupula present. Harpe trapezoidal in lateral view (proximoventral and distodorsal margins of harpe with projections in lateral view); shorter than gonostipes in lateral view, reaching 3/5 of gonostipes. Proximal end of dorsomedian conjunctiva of the gonostyle-volsella complex acute. Gonostyle-volsella complex with medioventral ridge present, gonostyle-volsella complex with length equal that of the gonostipes. Gonostipes longer than width; parossiculus separated from gonostipes; dorsal side of gonostipes flat. Parossiculus with one seta apically. Gonossiculus with two spines apically in lateral view. Penisvalva hyaline. S9 blunt, with double transverse row of five or six setae; submedial projections on proximal margin of S9 present. Distal margin of male S9 with projections. Proximolateral corner of male S9 with projections, but not acute. Proximal margin of S9 without projections. Medial projections on proximal margin of S9 present, length shorter than but >1/2 the length of S9 shield. S9 shield width: length = 1.6; distal part of the S9 shield darker brown, proximal part translucent and paler in color.

**Female**. Unknown.

##### Etymology.

The species is named after Jilong County, where the species was collected.

##### Distribution.

China (Xizang).

##### Remarks.

This species is similar to *Conostigmus lepus* Trietsch, 2020, but can be distinguished from *Conostigmus lepus* by the proximodorsal notch of the cupula being blunt or straight and the dorsal side of gonostipes is flat. In *Conostigmus lepus*, the proximodorsal notch of cupula is arched (inverted U-shaped), and the dorsal side of gonostipes is arched.

#### ﻿ Conostigmus shigatsensis

Taxon classificationAnimaliaHymenopteraMegaspilidae

Wang & Zhu sp. nov.

5BE89E04-1D74-5343-B3F5-AE46C6EC70B0

https://zoobank.org/17B96586-FD7E-4912-952D-962142168C06

Figs 7, 8

##### Material examined.

***Holotype***: • 1♂ (AHNU), **China**: Xizang, Jilong, Rikaze, 3 Aug. 2021, D. Wu leg., XZJL-8. ***Paratypes***: • 1♂ (AHNU), **China**: same collection information as preceding, XZJL-27. • 1♂ (AHNU), **China**: Xizang, Jilong, Rikaze, 17 Apr.-23 May. 2021, D. Wu leg., XZJL-16.

##### Diagnosis.

This new species can be separated from other *Conostigmus* species by the following characters: harpe of male genitalia trapezoidal in lateral view (flattened at the terminal; proximoventral margin of harpe with projections, distodorsal margin of harpe flat in lateral view), shorter than gonostipes in lateral view; gonossiculus with two spines apically. Cupula particularly short, only 1/10 the length of gonostyle-volsella complex in lateral view. Preoccipital furrow Preoccipital furrow present, not reaching posterior ocellar; notauli and median mesoscutal sulcus very distinct, grooved; anterior mesopleural sulcus distinct, with continuous fovea; mesometapleural sulcus distinct, with continuous fovea; pterostigma 3.2× as long as wide; sternaulus 0.8× as long as the mesopleuron.

##### Description.

**Male.** Body length: 1.28–1.29 mm.

**Figure 7.**
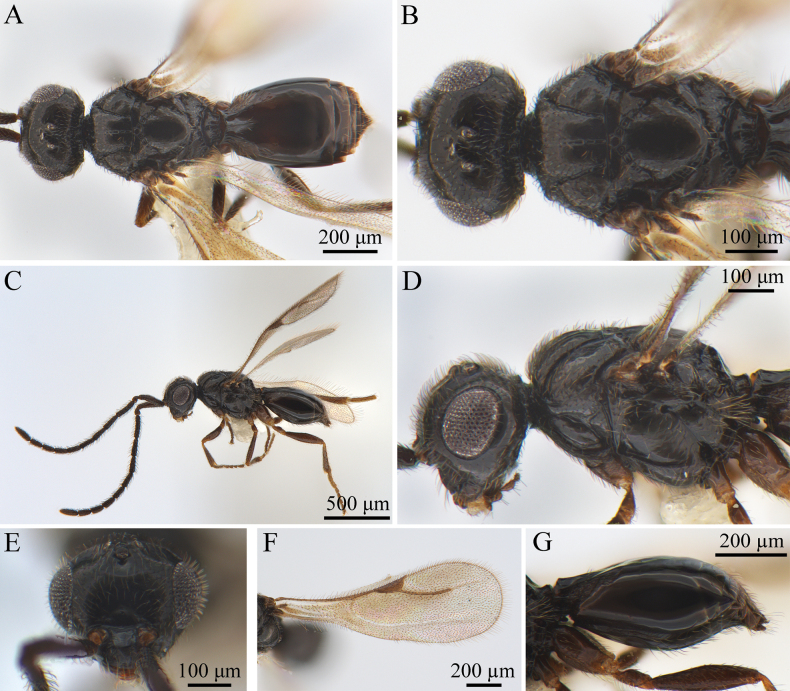
*Conostigmus shigatsensis* Wang & Zhu, sp. nov., male, holotype. **A.** Dorsal habitus; **B.** Head and mesosoma, dorsal view; **C.** Lateral habitus; **D.** Head and mesosoma, lateral view; **E.** Head, anterior view; **F.** Wings; **G.** Metasoma, lateral view.

***Coloration*** (Fig. 7C). Head, mesosoma, and metasoma black. Scape, pedicel, and flagellum black. Mouthparts brown; eyes silvery; ocelli silvery-black. Legs usually brown, sometimes black proximally, especially on middle sides of femora and tibiae. Syntergum black. Pterostigma and stigmal vein pale brown. Body pubescence white; marginal fringes of wings brown.

***Antennae*** (Fig. 7C). Scape ~3 × longer than wide, pedicel small and almost a droplet-shape. Male scape length vs pedicel length: 3.2–4.6. Scape length vs F1 length: 1.2. F1 length vs F2 length: 1.1–1.2. F1 longest flagellomere; F6, F7, and F8 shortest and equal in length. Setae lengths equal to flagellomere width.

***Head*** (Fig. 7D, E). Head width same as mesosoma. HH: HL = 1.4–1.5. HW: IOS = 1.6. HW: HH = 1.1. POL: OOL = 0.6–0.7. Ocellar triangle with short base, OOL: LOL = 2.6–2.8. Head circular in anterior view. Facial pit present, facial sulcus absent. Preocellar pit present, ocellar fovea present. Preoccipital lunula present. Preoccipital furrow present, not reaching anterior ocellar. Preoccipital carina present. Upper margins of scrobes straight, intertorular carina absent. Head with dense hairs.

***Mesosoma*** (Fig. 7B, D). Mesosoma narrow, 1.3× longer than wide. Length/width/height = 480/360/340 µm; densely pubescent, alutaceous in sculpture; mesoscutum length/width = 150/360 µm, mesoscutum 2.4× wider than long, Ascw/Pscw = 260/270 µm; notauli and median mesoscutal sulcus very distinct, groove; scutellum longer than mesoscutum, length/width = 210/190 µm; scutoscutellar sulcus foveolate, single fovea of the scutoscutellar sulcus length equal to width, continuous with interaxillar sulcus. Axilla width longer than length. Pronotum triangular, with an extra groove. Anterior mesopleural area present; anterior mesopleural sulcus distinct; posteroventral area (part of mesopleuron) smooth, with sparse setae; mesometapleural sulcus distinct, groove, in contact with mesopleural pit, mesopleural pit expansion; ventral division of metapleuron smooth, with sparse setae; pleural carina with long bristles. Sternaulus present, 0.8× as long as the mesopleuron. Anteromedian projection of the metanoto-propodeo-metapecto-mesopectal complex present.

***Wings*** (Fig. 7F). Forewing length 1.3 mm, with pterostigma, radius, and some transparent veins. Hyaline, densely pubescent and marginal fringes numerous. Pterostigma length/width = 170/50 µm, 3.2× as long as wide, triangular, posterior margin (part of pterostigma) curve. Stigmal vein 290 µm, slightly curved in the latter section and 1.7× longer than the pterostigma.

***Metasoma*** (Fig. 7G). Metasoma 1.8× longer than wide, length/width/height = 600/340/260 µm. Syntergum smooth, reaching 7/10 of metasoma length. Syntergum with three distinct gastral carinae, reaching 1/4 of syntergum length. Syntergal translucent patch present, elliptical.

**Figure 8.**
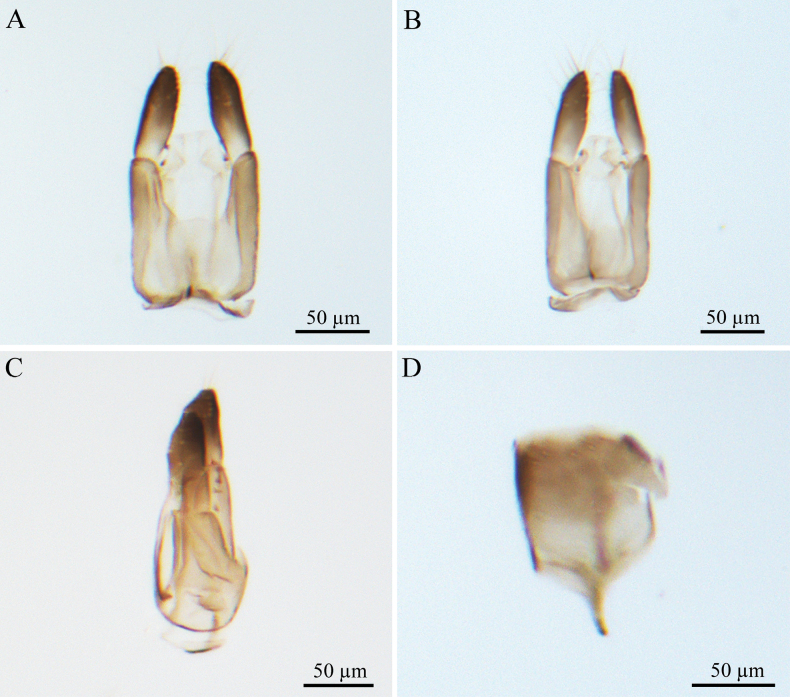
*Conostigmus shigatsensis* Wang & Zhu, sp. nov., male, holotype, genitalia. **A.** Dorsal view; **B.** Ventral view; **C.** Lateral view; **D.** S9.

***Male genitalia*** (Fig. 8). Genitalic cupula present, short, proximodorsal notch of cupula blunt, without a darker brown coloration near the central region; distodorsal margin of cupula straight. Cupula length vs gonostyle-volsella complex length: cupula particularly short, only 1/10 the length of gonostyle-volsella complex in lateral view. Proximoventral median projection of cupula absent. Harpe trapezoidal in lateral view (flatten at the terminal; proximoventral margin of harpe with projections, distodorsal margin of harpe flat in lateral view); shorter than gonostipes in lateral view, reaching 2/3 of gonostipes; harpe with more than 14 setae. Proximal end of dorsomedian conjunctiva of the gonostyle-volsella complex blunt. Gonostipes longer than wide; parossiculus separated from gonostipes. Gonostyle-volsella complex with medioventral ridge absent, gonostyle-volsella complex with length half that of the gonostipes. Parossiculus with one seta apically. Gonossiculus with two spines apically in lateral view. Penisvalva hyaline. S9 blunt, with double transverse row of four or five setae; submedial projections on proximal margin of S9 absent. Distal margin of male S9 concave. Proximolateral corner of male S9 with acute projections. Proximal margin of S9 with projections. Medial projections on proximal margin of S9 present, length <1/2 the length of S9 shield. Distal part of S9 shield darker brown, proximal part translucent and paler in color.

**Female.** Unknown.

##### Etymology.

The species is named after Shigatse City, where the species was collected.

##### Distribution.

China (Xizang).

##### Remarks.

This species is similar to *C. abdominalis* but can be distinguished by the facial sulcus being absent (present in *C. abdominalis*).

#### ﻿ Conostigmus kairus

Taxon classificationAnimaliaHymenopteraMegaspilidae

Wang & Zhu sp. nov.

D7F1789B-2743-55C5-BB3E-C984C8A180AF

https://zoobank.org/E34E4448-56B7-46AD-998E-A293C8ED7969

Figs 9, 10

##### Material examined.

***Holotype***: • 1♂ (AHNU), **China**: Xizang, Jilong, Rikaze, 17 Apr.-23 May. 2021, D. Wu leg., XZL-7. ***Paratypes***: • 4♂♂ (AHNU), **China**: same collection information as preceding, XZL-8, XZL-9, XZL-5, XZL-3; • 1♂ (AHNU), **China**: Xizang, Jilong, Rikaze, 3 Aug. 2021, D. Wu leg., XZS-18; • 1♂ (AHNU), **China**: Xizang, Jilong, Rikaze, 20 May. 2021, D. Wu leg., XZYG-6.

##### Diagnosis.

This new species can be separated from other *Conostigmus* species by the following characters: harpe of male genitalia columnar in lateral view (proximodorsal margin of harpe concave, Medioventral margin of harpe with projections in lateral view), equal to gonostipes length in lateral view; gonossiculus with two spines apically. Proximodorsal notch of cupula blunt or straight, with a darker brown coloration near the central region. Proximodorsal notch of cupula blunt or straight. Preoccipital furrow reaching the line connecting the posterior ocellar, not reaching anterior ocellus; notauli and median mesoscutal sulcus very distinct, grooved; anterior mesopleural sulcus distinct, with continuous fovea; mesometapleural sulcus distinct, with continuous fovea; pterostigma 3.6× as long as wide; sternaulus equal to mesopleuron length at level of sternaulus; hindwing with one transparent vein.

##### Description.

**Male.** Body length: 1.5–1.9 mm.

**Figure 9.**
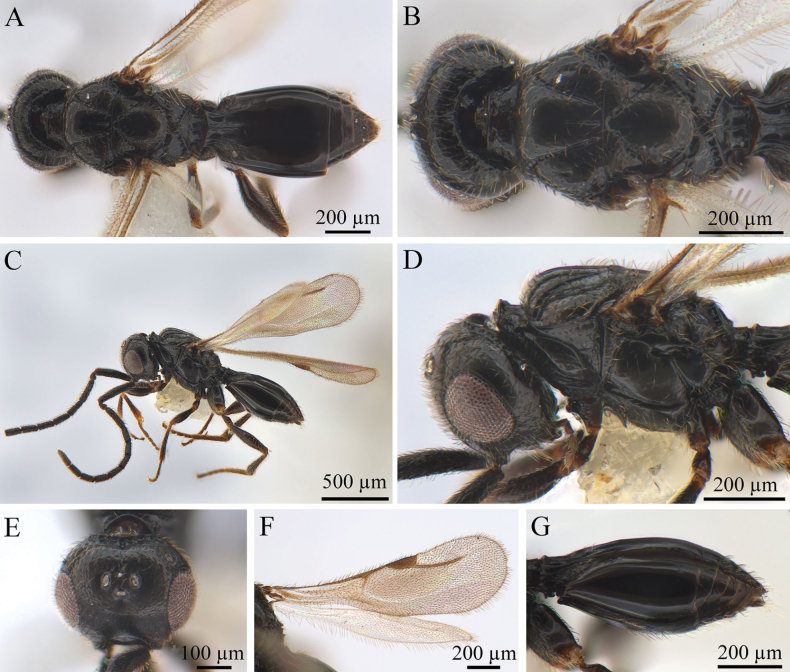
*Conostigmus kairus* Wang & Zhu, sp. nov., male, holotype. **A.** Dorsal habitus; **B.** Head and mesosoma, dorsal view; **C.** Lateral habitus; **D.** Head and mesosoma, lateral view; **E.** Head, anterior view; **F.** Wings; **G.** Metasoma, lateral view.

***Coloration*** (Fig. 9C). Head, mesosoma, and metasoma black. Scape, pedicel, and flagellum black. Mouthparts brown; eyes silvery; ocelli silvery-black. Legs usually brown, sometimes black proximally, especially on middle sides of femora and tibiae. Syntergum black. Pterostigma and vein pale brown. Body pubescence white; marginal fringes of wings brown.

***Antennae*** (Fig. 9C). Scape ~3 × longer than wide, pedicel small and droplet-shaped. Male scape length vs pedicel length 4.6–5.0. Scape length vs F1 length 1.2–1.5. F1 length vs F2 length 1.2–1.3. F1 longest. F7 and F8 shortest, equal in length. Setae short, not reaching 1/3 of flagellomere width.

***Head*** (Fig. 9D, E). Head width same as mesosoma. HH: HL = 1.3–1.4. HW: IOS = 1.6. HW: HH = 1.0–1.1. POL: OOL = 0.6–0.8. Ocellar triangle with short base, OOL: LOL = 2.4–2.8. Head circular in anterior view. Facial pit present, facial sulcus absent. Preocellar pit present, ocellar fovea present. Preoccipital lunula present. Preoccipital furrow reaching the line connecting the posterior ocellar, not reaching anterior ocellus. Preoccipital carina present. Upper margins of scrobes straight, intertorular carina absent. Head with dense hairs.

***Mesosoma*** (Fig. 9B, D). Mesosoma narrow, 1.3× longer than wide: length/width/height = 540/420/340 µm; densely pubescent, alutaceous in sculpture; mesoscutum length/width = 200/360 µm, mesoscutum 1.8× wider than long, Ascw/Pscw = 250/280 µm; notauli and median mesoscutal sulcus very distinct, groove; scutellum width equal to length, 210 µm; scutoscutellar sulcus foveolate, single fovea of the scutoscutellar sulcus length longer than width, continuous with interaxillar sulcus. Axilla width longer than length. Pronotum triangular, with an extra groove. Anterior mesopleural area present; anterior mesopleural sulcus distinct, grooved; posteroventral area (part of mesopleuron) smooth, with sparse setae; mesometapleural sulcus distinct, grooved, in contact with mesopleural pit, mesopleural pit present; ventral division of metapleuron smooth, with sparse setae; pleural carina with long bristles. Sternaulus present, sternaulus and mesopleuron are equal in length. Anteromedian projection of the metanoto-propodeo-metapecto-mesopectal complex present.

***Wings*** (Fig. 9F). Forewing length 1.4 mm, with pterostigma, stigmal vein. Hyaline, densely pubescent and marginal fringes numerous. Pterostigma length/width = 180/50 µm, 3.6× as long as wide; triangular, posterior margin (part of pterostigma) straight. Stigmal vein 250 µm, slightly curved in the latter section and 1.4× longer than pterostigma.

***Metasoma*** (Fig. 9G). Metasoma 1.9× longer than wide: length/width/height = 600/340/260 µm. Syntergum smooth, reaching 7/10 of metasoma length. Syntergum with three distinct gastral carinae, reaching 1/3 of syntergum length. Syntergal translucent patch present, elliptical.

**Figure 10.**
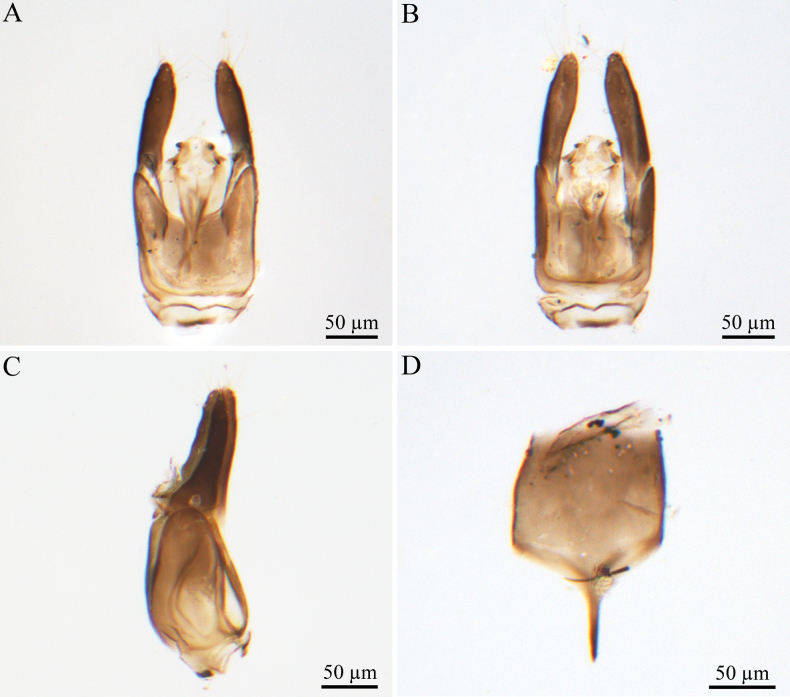
*Conostigmus kairus* Wang & Zhu, sp. nov., male, holotype, genitalia. **A.** Dorsal view; **B.** Ventral view; **C.** Lateral view; **D.** S9.

***Male genitalia*** (Fig. 10). Genitalic cupula present, proximodorsal notch of cupula blunt or straight, with a darker brown coloration near the central region; distodorsal margin of cupula straight. Harpe columnar in lateral view (proximodorsal margin of harpe concave, medioventral margin of harpe with projections in lateral view); length equal to gonostipes length in lateral view; harpe with more than six setae. Proximal end of dorsomedian conjunctiva of the gonostyle-volsella complex blunt. Cupula length vs gonostyle-volsella complex length: cupula short, <1/2 the length of gonostyle-volsella complex in lateral view. Proximoventral median projection of cupula present. Gonostipes longer than width; parossiculus separated from gonostipes. Parossiculus with one seta apically. Gonostyle-volsella complex with medioventral ridge absent. Gonossiculus with two spines apically in lateral view. Penisvalva hyaline. S9 blunt, eight setae arranged irregularly; submedial projections on proximal margin of S9 absent. Distal margin of male S9 flat. Proximolateral corner of male S9 with projections, not acute. Medial projections on proximal margin of S9 present, length equal to 1/2 the length of S9 shield.

**Female.** Unknown.

##### Etymology.

The new species is named after the Kaire waterfall, a well-known cultural icon in the area where it was collected.

##### Distribution.

China (Xizang).

##### Remarks.

This species is similar to *Conostigmus dimidiatus* (Thomson, 1858), but can be distinguished by the following characteristics: in *Conostigmus kairus*, the harpe is equal in length to the gonostipes in lateral view, and the proximodorsal notch of the cupula is blunt or straight, whereas in *C. dimidiatus*, the harpe is shorter than gonostipes in lateral view, and the proximodorsal notch of cupula is arched (inverted U-shaped).

#### ﻿ Dendrocerus

Taxon classificationAnimaliaHymenopteraMegaspilidae

Ratzeburg, 1852

0F8867BF-04BE-5EF2-96CA-BAA9AC7B5C1B

Dendrocerus Ratzeburg, 1852: 180.
Lygocerus Förster,1856: 97.
Macrostigma Rondani,1877: 184.
Atritomus Förster,1878: 56.
Prodendrocerus Kieffer, 1907: 11.
Atritomellus Kieffer,1914: 69.
Neolygocerus Muesebeck & Walkley, 1956: 374.
Basoko Risbec, 1958: 111.

##### Diagnosis.

Antennae 11-segmented in both sexes. Male flagellomeres asymmetrical in most cases, sometimes symmetric, segments 1–6 often branched or serrated (trapezoidal or triangular). POL longer than OOL. Facial sulcus absent. Wings present; pterostigma present. Notauli sharply angulate anteriorly; anteromedian projection of the metanoto-propodeo-metapecto-mesopectal complex present (not bifurcated) or absent. Male parossiculi fused, and each parossiculus with gonostipes not fused. Sternaulus absent.

#### ﻿ Dendrocerus sergii

Taxon classificationAnimaliaHymenopteraMegaspilidae

Alekseev, 1994

64E213DB-8CC1-5F9A-B202-C12962905EFB

Figs 11, 12

Dendrocerus sergii Alekseev, 1994: 153.

##### Species notes and history.

*Dendrocerus sergii* was first described by Alekseev in 1994, who described the male antennae, wings, head, mesosoma, and metasoma and illustrated the antennae for the first time. However, the morphological characteristics were represented by hand-made drawings, without records of the male genitalia or images of the female head, mesosoma, or metasoma. The present paper redescribes the male, adding descriptions of the male genitalia and color photographs. Furthermore, this article is the first to describe the syntergal translucent patch and S9.

##### Material examined.

• 2♂♂ (AHNU), **China**: Xizang, Jilong, Rikaze, 17 Apr.–23 May. 2021, D. Wu leg., XZL-2, XZL-1; • 1♂ (AHNU), **China**: Xizang, Jilong, Rikaze, 10–20 May. 2021, D. Wu leg., XZKJQ-2; • 1♂ (AHNU), **China**: Xizang, Jilong, Rikaze, 3 Aug. 2021, D. Wu leg., XZQ-1.

##### Diagnosis.

This species can be separated from other *Dendrocerus* species by the following characters: harpe of male genitalia triangular in lateral view (terminally flat in lateral view; distoventral margin of harpe with projections, distodorsal margin of harpe concave in lateral view in other species), rectangular in dorsal and ventral views, gonossiculus with two setae apically. Gonostyle–volsella complex with medioventral ridge incomplete, length equal that of the gonostyle–volsella complex. Facial pit present, preoccipital furrow grooved, not reaching anterior ocellar; anterior mesopleural sulcus present; posteroventral area (part of mesopleuron) smooth; mesometapleural sulcus present, grooved, not in contact with mesopleural pit.

##### Description.

**Male.** Body length: 1.5–2.2 mm.

**Figure 11.**
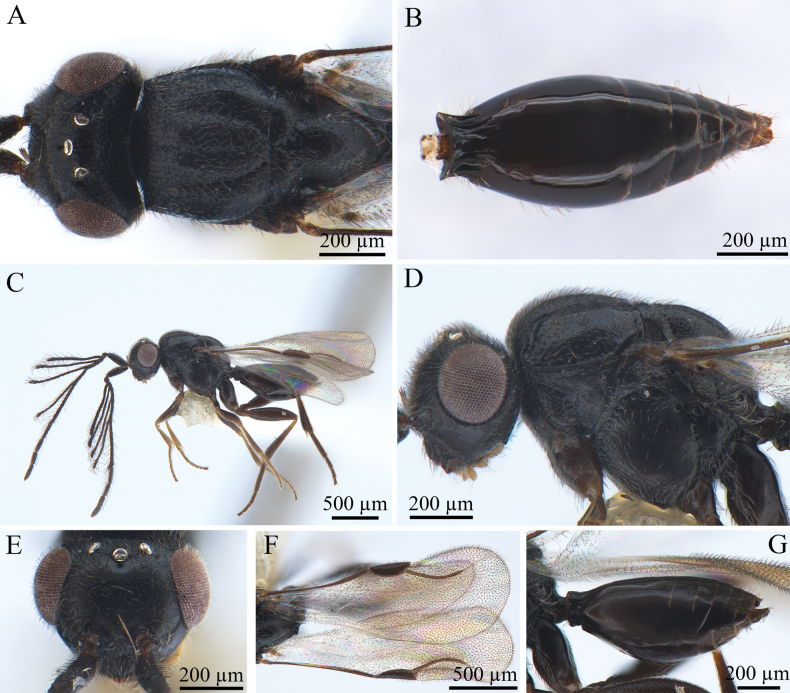
*Dendrocerus sergii* Alekseev, 1994, male. **A.** Head and mesosoma, dorsal view; **B.** Metasoma, dorsal view; **C.** Lateral habitus; **D.** Head and mesosoma, lateral view; **E.** Head, anterior view; **F.** Wings; **G.** Metasoma, lateral view.

***Coloration*** (Fig. 11C). Head, mesosoma, and metasoma black. Flagellum dark brown, scape and pedicel brown. Mouthparts brown; eyes silvery; ocelli silvery-black. Legs usually brown, sometimes darkened proximally, especially on femora and tibiae. Pterostigma and stigmal vein dark brown. Body pubescence white; marginal fringes of wings brown.

***Antennae*** (Fig. 11C). Ramose with five branches; scape ~3 × longer than wide, pedicel small and rounded. Male scape length vs pedicel length 3.3–3.8. Scape length vs F1 length 5.4–6.0. F1 length vs F2 length 0.6–1.0. F5 longest flagellomere, F8 shortest. A1 length vs F1 length 12.2–15.8. A2 length vs F2 length 10.7–11.8. A3 length vs F3 length 5.2–6.7. A4 length vs F4 length 2.7–3.0. A5 short, A5 length vs F5 length 0.17–0.22. Setae long, reaching 2–4× flagellomere width.

***Head*** (Fig. 11B, E). Head width 1.1× longer than mesosoma width. HH: HL = 0.8–1.6. HW: IOS = 1.7–1.8. HW: HH = 1.2–1.6. POL: OOL = 1.9–2.0. Ocellar triangle with long base, OOL: LOL = 1.3–1.4. Head circular in anterior view. Facial pit present and shallow, facial sulcus absent. Preocellar pit present, ocellar fovea present. Preoccipital lunula present. Preoccipital furrow present, grooved, not reaching posterior ocellar. Preoccipital carina present. Scrobes absent, intertorular carina absent. Head with sparse hairs.

***Mesosoma*** (Fig. 11A, D). Mesosoma slightly narrow, 1.6× longer than wide, length/width/height = 770/490/590 µm; densely pubescent, alutaceous in sculpture; mesoscutum length/mesoscutum width = 400/490 µm, mesoscutum 1.2× wider than long, Ascw/Pscw = 400/370 µm; notauli and median mesoscutal sulcus very distinct, notauli groove, median with continuous fovea, fovea deep, equal to or longer than width; shallow sulcus on the mesoscutum lying lateral to the notaulus and parallel to median mesoscutal sulcus, 1/2 length of mesoscutum. Scutellum length/scutellum width = 300/340 µm, scutellum width almost equal to length; scutoscutellar sulcus foveolate; fovea (part of scutoscutellar sulcus) length shorter than width, continuous with interaxillar sulcus. Axilla width slightly longer than length. Posterior of scutellum foveolate. Pronotum triangular, anterior mesopleural area present; anterior mesopleural sulcus present; posteroventral area (part of mesopleuron) smooth; mesometapleural sulcus present, groove, not contact with mesopleural pit, mesopleural pit expansion; ventral division of metapleuron densely pubescent; pleural carina with long bristles. Anteromedian projection of the metanoto-propodeo-metapecto-mesopectal complex absent.

***Wings*** (Fig. 11F). Forewing length 1.9 mm, with pterostigma, radius, and some transparent veins. Hyaline, densely pubescent, and marginal fringes numerous. Stigma length/width = 270/100 µm, 2.7× as long as wide, semicircular. Stigmal vein 440 µm, slightly curved in the latter section and 1.6× longer than pterostigma.

***Metasoma*** (Fig. 11G). Metasoma 2.5× longer than wide, length/width/height = 950/380/390 µm. Syntergum smooth, reaching 1/2 of metasomal length. Syntergum with six distinct gastral carinae, reaching 1/4 of syntergum length. Syntergal translucent patch present, orbicular.

**Figure 12.**
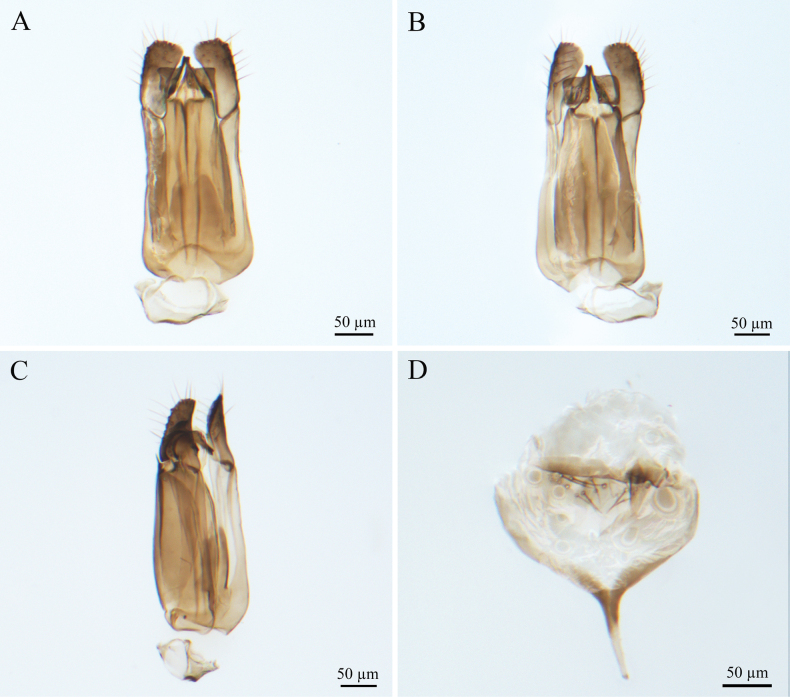
*Dendrocerus sergii* Alekseev, 1994, male, genitalia. **A.** Dorsal view; **B.** Ventral view; **C.** Lateral view; **D.** S9.

***Male genitalia*** (Fig. 12). Genitalic cupula present, proximodorsal notch of cupula blunt or straight, with a darker brown coloration near the central region; distodorsal margin of cupula straight. Cupula length vs gonostyle–volsella complex length: cupula short, only 1/4 the length of gonostyle–volsella complex in lateral view. Proximoventral median projection of cupula present. Harpe triangular in lateral view, with a flat terminus, a distoventral margin bearing projections, and a concave distodorsal margin; rectangular in dorsal view; length shorter than gonostipes in lateral view, reaching 1/2 of gonostipes. Proximal end of dorsomedian conjunctiva of the gonostyle–volsella complex acute. Gonostipes longer than width; parossiculus separated from gonostipes. Gonostyle–volsella complex with medioventral ridge incomplete, its length equal that of the gonostyle–volsella complex. Parossiculus with one seta apically. Gonossiculus rectangular in ventral view, with two setae apically. Penisvalva straight. S9 circular, with irregular row of setae and more than 13 setae; submedial projections on proximal margin of S9 absent; distal margin of male S9 flat; proximolateral corner of male S9 with projections, not acute; proximal margin of S9 without projections. Medial projections on proximal margin of S9 present, length of projections 1/3 length of S9 shield.

##### Distribution.

China (Xizang) – new record for China, Vietnam.

#### ﻿ Dendrocerus tumidulus

Taxon classificationAnimaliaHymenopteraMegaspilidae

Wang & Zhu sp. nov.

9A2B4DED-5190-5C32-AF6F-7A0CF3D8C874

https://zoobank.org/BBEA16E3-DD16-4AA4-8B4C-56764206D9CD

Figs 13, 14

##### Material examined.

***Holotype***: • 1♂ (AHNU), **China**: Xizang, Jilong, Rikaze, 17 Apr. 2021, D. Wu leg., XZ-32. ***Paratypes***: • 4♂♂ (AHNU), **China**: same collection information as holotype, XZS-1, XZJL-4, XZ-33, XZJL-3, XZ-32; • 3♂♂ (AHNU), **China**: Xizang, Jilong, Rikaze, 20 May. 2021, D. Wu leg., XZYG-2, XZYG-7, XZYG-3; • ♂ (AHNU), **China**: Xizang, Jilong, Rikaze, 21 Feb–1 May. 2021, D. Wu leg., XZS-14; • 3♂♂ (AHNU), **China**: Xizang, Jilong, Rikaze, 3 Aug. 2021, D. Wu leg., XZS-14, XZ-6, XZ-22.

##### Diagnosis.

This new species can be separated from other *Dendrocerus* species by the following characters: harpe of male genitalia triangular in lateral view (terminal point, distodorsal margin of harpe with projections, distoventral margin of harpe flat, in lateral view), strip-shaped in dorsal and ventral views, gonossiculus with two setae apically. Facial pit present, preoccipital furrow groove, not reaching anterior ocellar; anterior mesopleural sulcus present; posteroventral area (part of mesopleuron) smooth; mesometapleural sulcus present, grooved, not in contact with mesopleural pit; pterostigma 3.5× as long as wide; syntergum carinae absent; syntergal translucent patch orbicular.

##### Description.

**Male.** Body length: 1.0–1.2 mm.

**Figure 13.**
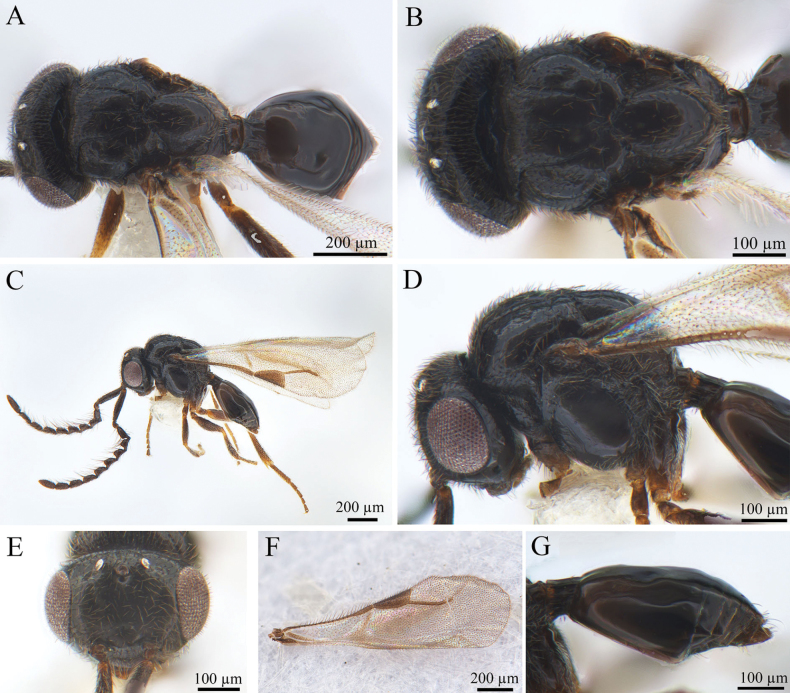
*Dendrocerus tumidulus* Wang & Zhu, sp. nov., male, holotype. **A.** Dorsal habitus; **B.** Head and mesosoma, dorsal view; **C.** Lateral habitus; **D.** Head and mesosoma, lateral view; **E.** Head, anterior view; **F.** Wings; **G.** Metasoma, lateral view.

***Coloration*** (Fig. 13C). Head, mesosoma, and metasoma black. Antennae black or brown. Mouthparts brown; eyes silvery or brown; ocelli silvery. Legs usually brown, sometimes darkened proximally, especially on femora and tibiae. Pterostigma and vein dark brown. Body pubescence white; marginal fringes of wings brown.

***Antennae*** (Fig. 13C). Scape ~4 × longer than wide, pedicel small and almost a droplet. Male scape length vs pedicel length: 4.5–4.7. Scape length vs F1 length: 2.0–2.9. F1 length vs F2 length: 1.1–1.3. Longest flagellomere: F9. Shortest flagellomere: F2. F1–F8 trapezoidal; length vs width: 2. Setae long, length reaching 2× flagellomere width.

***Head*** (Fig. 13C, E). Head width 1.2× longer than mesosoma width. HH: EHf = 1.4–1.9. HH: HL = 1.2–1.4. HW: IOS = 1.5–1.6. HW: HH = 1.2–1.3. POL: OOL = 1.3–1.5. Ocellar triangle with long base, OOL: LOL = 1.4–1.8. Head circular in anterior view. Facial pit present, facial sulcus absent. Preocellar pit present, ocellar fovea present (Fig. 13E). Preoccipital lunula present. Preoccipital furrow present, grooved, not reaching anterior ocellar. Preoccipital carina present. Upper margins of scrobes W-shaped, intertorular carina present (Fig. 13E). Head with sparse hairs.

***Mesosoma*** (Fig. 13B, D). Mesosoma slightly narrow, 1.5× longer than wide, length/width/height = 470/320/380 µm; densely pubescent, alutaceous in sculpture; mesoscutum length/mesoscutum width = 180/320 µm, mesoscutum 1.8× longer than wide, Ascw/Pscw = 260/260 µm; notauli and median mesoscutal sulcus very distinct, groove; continuous with interaxillar sulcus. Scutellum width almost equal to length (length/width = 300/340 µm); scutoscutellar sulcus foveolate; single fovea of the scutoscutellar sulcus length shorter than width, continuous with interaxillar sulcus. Axilla width slightly longer than length. Posterior of scutellum foveolate. Pronotum triangular, anterior mesopleural area present; anterior mesopleural sulcus present; posteroventral area (part of mesopleuron) smooth; mesometapleural sulcus present, groove, not in contact with mesopleural pit, mesopleural pit present; ventral division of metapleuron smooth; pleural carina with long bristles. Anteromedian projection of the metanoto-propodeo-metapecto-mesopectal complex absent.

***Wings*** (Fig. 13F). Forewing length 1.3 mm, with pterostigma, radius, and some transparent veins. Hyaline, densely pubescent, and marginal fringes numerous. Pterostigma (length/width = 210/60 µm) 3.5× as long as wide, semicircular, posterior margin (part of pterostigma) curve. Stigmal vein 230 µm, slightly curved in the latter and 1.1× longer than the stigma.

***Metasoma*** (Fig. 13G). Metasoma 1.6× longer than wide, length/width/height = 480/300/200 µm. Syntergum smooth, reaching 1/2 of metasomal length. Syntergum gastral carinae absent. Syntergal translucent patch present, orbicular.

**Figure 14.**
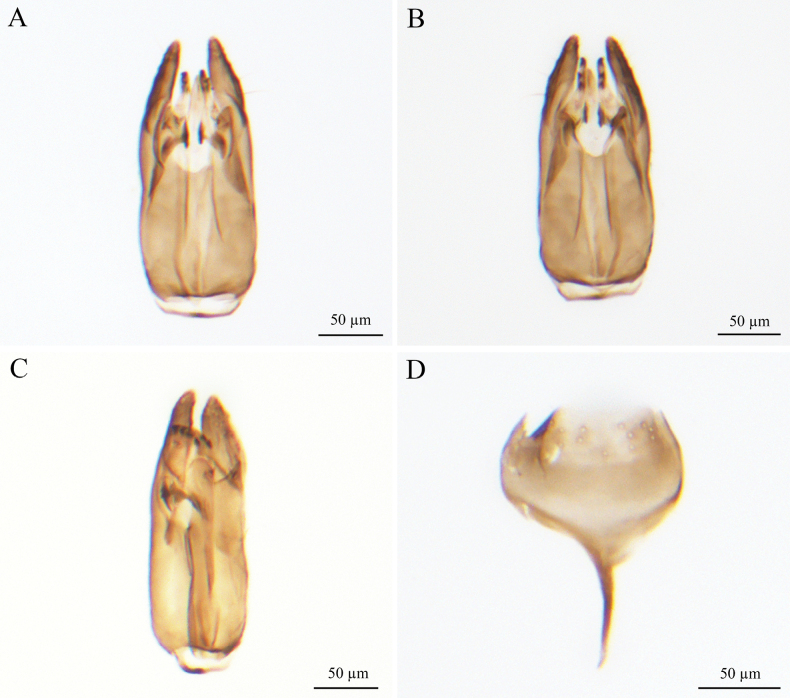
*Dendrocerus tumidulus* Wang & Zhu, sp. nov., male, holotype. **A.** Dorsal view; **B.** Ventral view; **C.** Lateral view; **D.** S9.

***Male genitalia*** (Fig. 14). Genitalic cupula present, proximodorsal notch of cupula straight, with a darker brown coloration near the region; distodorsal margin of cupula straight. Cupula length vs gonostyle–volsella complex length: cupula particularly short, only 1/10 the length of gonostyle–volsella complex in lateral view. Proximoventral median projection of cupula absent. Harpe triangular in lateral view (terminal point, distodorsal margin of harpe with projections, distoventral margin of harpe flat, in lateral view), strip-shaped in dorsal view or ventral view; shorter than gonostipes in lateral view, reaching 1/2 of gonostipes. Proximal end of dorsomedian conjunctiva of the gonostyle-volsella complex blunt. Gonostipes longer than width; parossiculus not separated from gonostipes. Gonostyle-volsella complex with medioventral ridge absent, gonostyle-volsella complex with length reaching 4/5 that of the gonostipes. Parossiculus with one seta apically. Gonossiculus with two spines apically in lateral view. Penisvalva hyaline, straight. S9 blunt, with three rows of setae and >12 setae; submedial projections on proximal margin of S9 absent; distal margin of male S9 flat; proximolateral corner of male S9 with projections, not acute. Proximal margin of S9 without projections. Submedial projections on proximal margin of S9 present, their lengths equal to the length of S9 shield.

**Female.** Unknown.

##### Etymology.

The new species is named after its characteristic mesosoma that is much higher than its metasoma.

##### Distribution.

China (Xizang).

##### Remarks.

This species is similar to *D. carpenteri* but differs in having F1–F8 trapezoidal (vs F1–F7 trapezoidal in *D. carpenteri*); it is also similar to *D. constrictus* but can be distinguished by the metasoma being equal in length to the mesosoma (longer in *D. constrictus*). Furthermore, it is similar to *D. laevis* but differs in possessing complete notauli on the mesoscutum (vs with incomplete notauli in *D. laevis*).

#### ﻿ Lagynodes

Taxon classificationAnimaliaHymenopteraMegaspilidae

Förster, 1841

F50103FF-E7AD-5B20-A3CB-49BE9675A74D

Microps Haliday, 1833: 272.
Lagynodes Förster, 1840: 46.
Triogmus Marshall, 1874: 134.
Plastomicrops Kieffer, 1906: 145.

##### Diagnosis.

Antennae 11-segmented in both sexes. Male flagellomeres symmetrical and cylindrical. Male ocellus present; wings present; pterostigma absent. Female ocellus present or absent; wing absent; anterior preoccipital carina (pc) absent and scrobes absent.

#### ﻿ Lagynodes acuticornis

Taxon classificationAnimaliaHymenopteraMegaspilidae

(Kieffer, 1906)

7418C6FB-7D36-52F2-9162-A9FD7A390134

Fig. 15

Plastomicrops acuticornis Kieffer, 1906: 145.
Lagynodes rautheri Wolff, 1918: 593.
Plastomicrops unicolor : Szelényi, 1936: 63.
Lagynodes acuticornis : Dessart, 1966: 37.
Lagynodes (Plastomicrops) acuticornis : Dessart, 1979: 262.

##### Material examined. •

2♀♀ (AHNU), **China**: Xizang, Jilong, Rikaze, 21 Feb.–20 May. 2021, D. Wu leg., XZ-10, XZS-11.

##### Diagnosis.

This species can be separated from other *Lagynodes* species by the following characters: F9 acutely pointed distally, F9 longest. F2 shortest; antennae setae short; head trapezoidal in anterior view, facial pit absent, facial sulcus absent. Preoccipital furrow absent; anterior preoccipital carina absent, scrobes absent, intertorular carina present, median process on intertorular carina absent; head with dense hairs; neck short; pronotum cylindrical, posterior margin concave; metamesosoma almost absent.

##### Description.

**Female.** Body length: 0.8–0.9 mm.

**Figure 15.**
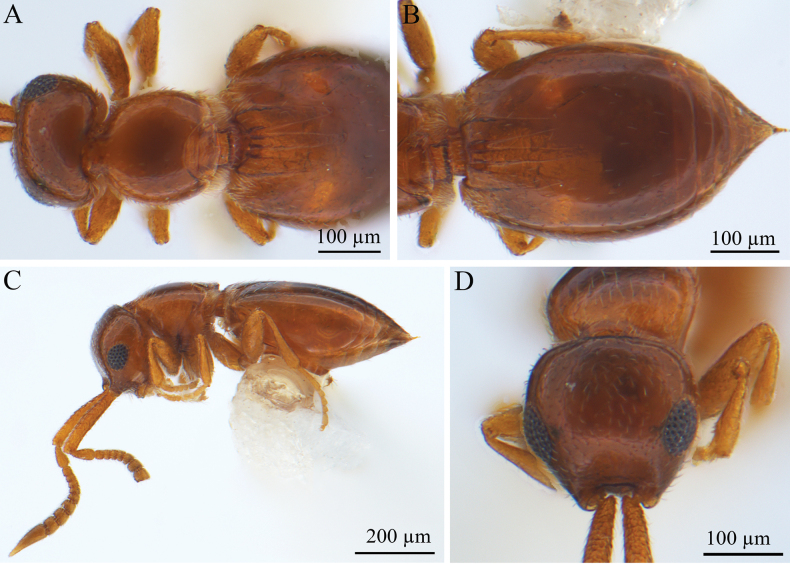
*Lagynodes acuticornis* (Kieffer, 1906), female. **A.** Dorsal habitus; **B.** Metasoma, dorsal view; **C.** Lateral habitus; **D.** Head, anterior view.

***Coloration*** (Fig. 15C). Head, mesosoma, and metasoma brown. Antennae brown. Mouthparts brown; eyes black. Legs brown. Body pubescence white.

***Antennae*** (Fig. 15C). Scape ~6 × longer than wide, pedicel small and almost droplet-shaped, F9 terminal sharp. Female scape length vs pedicel length: 3.6–3.7. Scape length vs F1 length: 2.5–2.6. F1 length vs F2 length: 1.0–1.2. F9 longest. F2 shortest. Setae short.

***Head*** (Fig. 15A, D). Head width longer than mesosoma width, head width vs mesosoma width 1.1–1.2. HH: HL = 0.7–0.8. HW: IOS = 1.4–1.5. HW: HH = 1.3–1.4. Ocellar absent. Head trapezoidal in anterior view. Facial pit absent. Preoccipital lunula absent. Preoccipital furrow absent. Preoccipital carina absent. Scrobes absent. Intertorular carina present. Median process on intertorular carina absent. Head with dense hairs.

***Mesosoma*** (Fig. 15A). Mesosoma narrow, 1.2× longer than wide; length/width/height = 230/180/130 µm; densely pubescent, alutaceous in sculpture; neck short; pronotum cylindrical, posterior margin concave; metamesosoma almost absent.

***Wings*.** Absent.

***Metasoma*** (Fig. 15B). Metasoma 1.6× longer than wide; length/width/height = 470/300/190 µm. Syntergum smooth, reaching 2/3 of metasomal. Syntergum with five distinct gastral carinae, reaching 2/5 of syntergum length. Syntergal translucent patch absent.

##### Distribution.

China (Xizang) – new record to China, Belgium, Canada, Finland, France, Germany, Mexico, Italy, Switzerland, Sweden, Turkey, United States, Ukraine.

##### Remarks.

This species is similar to *L. occipitalis* Kieffer, 1906, but can be distinguished by the length of preoccipital and eyes. *Lagynodes occipitalis* has a preoccipital >2× as long as the eyes; the metanotum and head are smooth. *Lagynodes acuticornis* has a preoccipital <2× as long as the eyes; the metanotum is rough.

## ﻿Discussion

**Figure 16.**
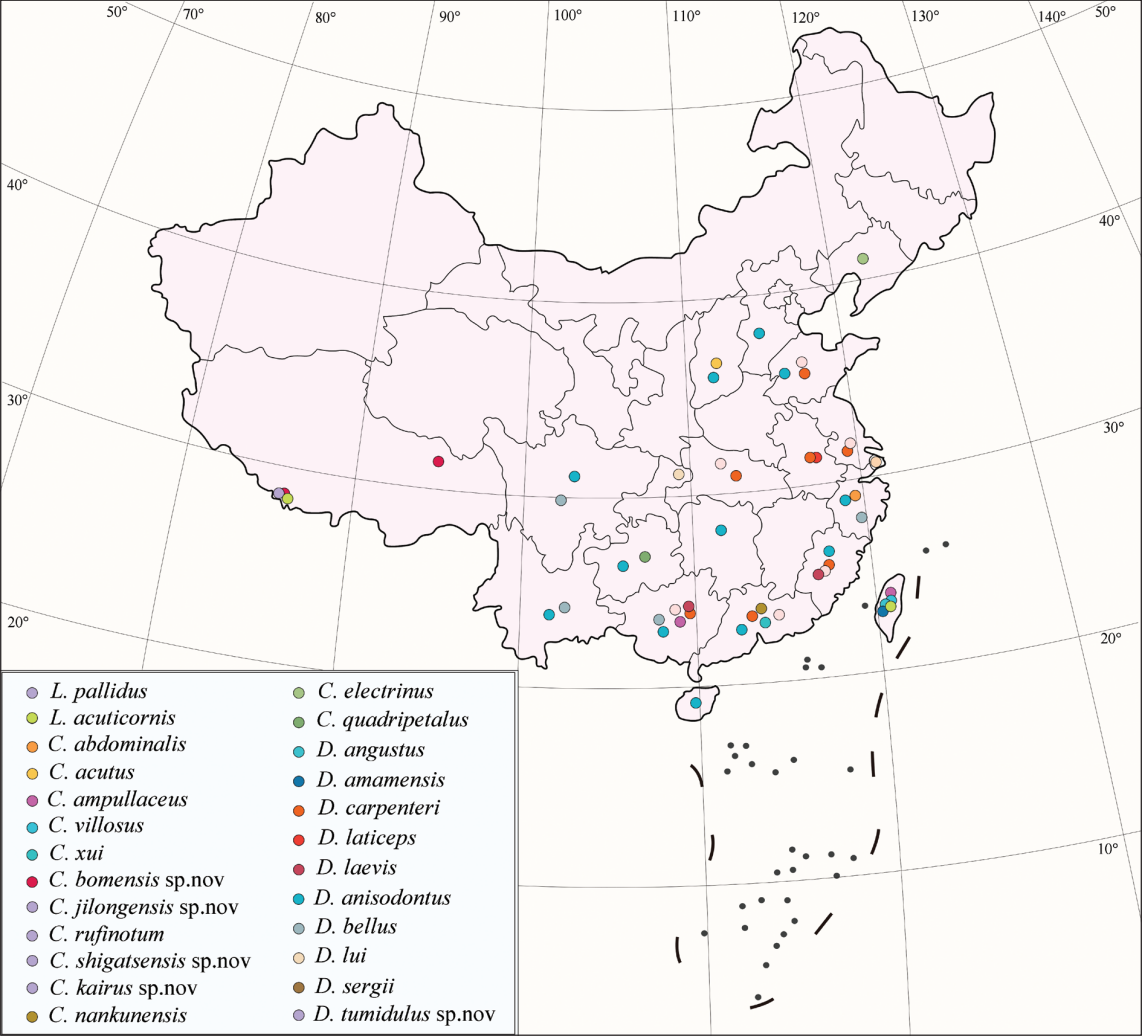
The distribution map of Chinese Megaspilidae species (location error: ± 50 km).

The family Megaspilidae is composed of two extant subfamilies. Megaspilinae is represented by 17 valid species recorded in China, while Lagynodinae is currently represented by one. There has been very little recent work on the Chinese Ceraphronoidea and it is generally a rather neglected group of parasitoids. In this study, we describe five new species and redescribe three known species, bringing the Chinese species number of Megaspilidae to 25 (Table 2, Fig. 16).

**Table 2.** List of Chinese species of Megaspilidae.

Species	Distribution	
	World	China
*L. pallidus* Boheman, 1832	NA, NO, OR, PA,	SW, EC
*L. acuticornis* (Kiffer, 1906)	NA, NO, PA	SW, EC
*C. abdominalis* Boheman, 1832	NA, PA	EC
*C. acutus* Wang & Chen, 2024	OR, PA	NC
*C. ampullaceus* Dessart, 1997	PA	EC
*C. bomensis* Wang & Zhu, sp. nov.	PA	SW
*C. electrinus* Wang & Chen, 2024	PA	NE
*C. nankunensis* Qian & Wang, 2024	PA	SC
*C. quadripetalus* Wang & Chen, 2024	PA	SW
*C. rufinotum* Dodd, 1914	AU, PA	SW
*C. jilongensis* Wang & Zhu, sp. nov.	PA	SW
*C. kairus* Wang & Zhu, sp. nov.	PA	SW
*C. shigatsensis* Wang & Zhu, sp. nov.	PA	SW
*C. villosus* Dessart, 1997	OR, PA	EC
*C. xui* Cui & Wang, 2023	PA	SC
*D. amamensis* Takada, 1974	PA	EC
*D. angustus* Dessart, 1999	AF, OR, PA	EC
*D. anisodontus* Wang, Chen & Mikó, 2021	PA	NC, HZ, EC, SW, SC
*D. bellus* Wang, Chen & Mikó, 2021	PA	SC, SW, EC
*D. carpenteri* Curtis, 1829	AU, NA, NO, OR, PA	EC, SC, HZ
*D. laevis* Ratzeburg, 1852	PA	EC, SC
*D. laticeps* Hedicke, 1929	AU, NA, OR, PA	EC, SC, HZ
*D. lui* Li & Wang, 2023	PA	SW
*D. sergii* Alekseev, 1994	OR, PA	SW
*D. tumidulus* Wang & Zhu, sp. nov.	PA	SW

*NA: Nearctic, NO: Neotropical (including all of Mexico and the Caribean), PA: Palaearctic, OR: Oriental (including all of China and India), AU: Australasian and Oceanian (including New Guinea and islands east), EC: East China, HZ: the Central of China, NC: North China, NE: Northeastern China, NW: Northwestern China, SC: Southern China, SW: Southwestern China.

The genus *Lagynodes* is unique within Megaspilidae because the females are wingless and completely lack the ocelli, which makes them immediately distinguishable from the unwinged species of the genera *Ceraphron* and *Megaspilus*. The males of *Lagynodes* are very similar to *Ceraphron*, but can be distinguished by the following characteristics: sharp, toothed, lamellar cutting edge between the root of the mid-leg [strongly compressed from the side (Förster, 1856)] and two spurs on the mid-leg. In this paper, we describe a newly recorded species of *Lagynodes* for China, but unfortunately, no male specimens were found in this study.

The morphologically based classification method makes it difficult to identify species to genus level. In *Dendrocerus* notauli are sharply angulate anteriorly, whereas in *Conostigmus* notauli are only slightly and steeply angulate anteriorly. However, in some *Conostigmus* species such as *Conostigmus linearis* Hellén, 1966, *Conostigmus laeviceps* Ashmead, 1893, *Conostigmus orcasensis* Brues, 1909, and *Conostigmus longiharpes* Trietsch, 2020, it becomes difficult to ascertain the genus based on the characteristics of their notauli. Generally, *Dendrocerus* are considered to have more rounded pterostigma, while *Conostigmus* have triangular pterostigma. Yet, exceptions exist: in *Conostigmus nigrorufus* Dessart, 1997 and *Conostigmus duncani* Trietsch, 2020, the pterostigma appear semi-circular (Alekseev and Radchenko 2001; Mikó et al. 2011; Trietsch et al. 2020).

In some instances, characters of the male genitalia serve as the sole means of distinguishing between *Conostigmus* and *Dendrocerus*. In all *Dendrocerus* species, the parossiculi are consistently fused with the gonostipes. Conversely, in *Conostigmus*, the parossiculi are typically independent, although some species, such as *Conostigmus dessarti* Trietsch & Mikó, 2020, *Conostigmus minimus* Trietsch & Mikó, 2020, *Conostigmus laeviceps* Ashmead, 1893, *Conostigmus musettiae* Trietsch & Mikó, 2020, *Conostigmus bipunctatus* Kieffer, 1907 and *Conostigmus franzinii* Trietsch & Mikó, 2020, may exhibit fused parossiculi (Mikó et al. 2011; Trietsch et al. 2020).

## Supplementary Material

XML Treatment for Conostigmus

XML Treatment for Conostigmus rufinotum

XML Treatment for Conostigmus bomensis

XML Treatment for Conostigmus jilongensis

XML Treatment for Conostigmus shigatsensis

XML Treatment for Conostigmus kairus

XML Treatment for Dendrocerus

XML Treatment for Dendrocerus sergii

XML Treatment for Dendrocerus tumidulus

XML Treatment for Lagynodes

XML Treatment for Lagynodes acuticornis

## ﻿Additional information

### Use of AI

No use of AI was reported.

### Author ORCIDs

Xu Wang https://orcid.org/0000-0001-6465-4052

Fang Li https://orcid.org/0009-0004-9269-7200

Wen-Jing Zhao https://orcid.org/0009-0009-8199-8442

Yi-Xin Huang https://orcid.org/0000-0002-7885-321X

Chun-Hui Xiang https://orcid.org/0009-0008-5840-3458

Hua-Yan Chen https://orcid.org/0000-0002-0382-1635

Chao-Dong Zhu https://orcid.org/0000-0002-9347-3178

### Data availability

All of the data that support the findings of this study are available in the main text.
